# Supplementary material for: Associations between the Gut Microbiota, Race, and Ethnicity of Patients with Colorectal Cancer: A Pilot and Feasibility Study
Source: Cancers (Basel). 2023 Sep 13;15(18):4546. doi: 10.3390/cancers15184546 (PMC10526839; doi:10.3390/cancers15184546)
Supplement: Supplementary file 1 [file cancers-15-04546-s001.zip › Supplementary Figure S1.pdf]

Supplementary Figure S1:

Microbiome Disparities Recruitment Script

Purpose of this document

This document is to be used when contacting participants to gauge interest in participating in the microbiome disparities study.

STEP 1 | INITIAL CONTACT IF BY TELEPHONE

If caller other than desired person: Hello - I am first name and surname from UCSF/ZSFGH. May I please speak to insert potential participant's name.

**When desired person is on the phone**	Hello [Mr/Mrs]_________ My name is First Name and I am a research coordinator calling from the division of Oncology at UCSF [if at ZSFGH: "Oncology clinic at San Francisco General"]<br><br>I am calling you to talk to you about participating in a research study. Is now an ok time for you to speak?<br><br>**IF NO, GO TO STEP 2**<br>**IF YES, GO TO STEP 5**
**If desired person is not available**	Is there a better day and time to reach [Mr/Mrs]_________?<br>Note days and times and enter into excel tracker<br>Thank you for your assistance. I will try to call back then.
**If answering machine**	Leave the following message: This is ___calling from UCSF [or ZSFGH] about a potential research study. If you are interested in hearing more, please give me a call back at ____

STEP 2 | Re-scheduling

**If interrupted or strong immediate refusal**	Is there another time I can call you back?<br><br>**IF YES**, Thank you. I will try to call back then. Would you be interested in reviewing a sample consent form for the study prior to the call?<br><br>**IF NO/NOT INTERESTED**. Okay. Thank you for your time. **End Call.**<br>Note and enter into RedCap

STEP 3 | INITIAL CONTACT IF IN PERSON

	Hello [Mr/Mrs]_________ My name is First Name and I am research coordinator at UCSF/ZSFGH (select one).<br><br>I would like to talk to you about participating in a research study. Is now an ok time to discuss it?<br><br>**IF NO, GO TO STEP 4**<br>**IF YES, GO TO STEP 5**

STEP 4 | Re-scheduling in person

**If immediate refusal**	Is there another time I can meet with you to discuss the study?<br><br>**IF YES**, Thank you. I will try arrange to meet with you then.<br><br>**IF NO/NOT INTERESTED**. Okay. Thank you for your time.

STEP 5 | STUDY DESCRIPTION

	You're eligible to be in this study because you are a patient in the UCSF GI Oncology Clinic/ZSFGH Oncology Clinic [select one]<br>We are conducting a study to learn more about the reasons why people get colon or rectal cancer. The information we get from this research may help us find new ways to prevent colon and rectal cancer.<br><br>If patient is African American, ask: In particular we are interested in learning why African Americans have higher rates of colon and rectal cancer than other racial groups.<br><br>Would you like to learn more about the study?<br>**YES**: Continue below<br>**NO:** I understand. Could I ask why you are not interested hearing more? (after explanation, say "Thank you for your time today"<br><br>If you decide to participate in this study, you will first be asked to complete a survey that asks about your diet and medical history. You can fill out the survey in a way that is convenient for you: in clinic, on paper or on a computer with research coordinator, or at home on your personal device. After completing the survey, you will be given a stool collection kit to take home and mail back a stool to us. You will be given a $25 gift card for your participation.<br><br>If you decide not to participate in this study, it will not change the care you receive.<br><br>Do you have any questions about the study?<br><br>Are you interested in participating?<br><br>If you'd like to participate, we can go ahead and schedule a time for me to meet with you to give you more information and review the consent paper work.<br><br>If you need more time to decide if you would like to participate, you may also call or email me with your decision.<br><br>**If interested in participating:** Schedule time and method for formal consent<br><br>**If not interested in participating:** I understand. Is there a reason you are not interested? (after explanation, say "Thank you for your time today"

STEP 6 | ENDING THE VISIT OR CALL

	Thank you again for your time. Do you have any questions?

	Again, my name is first name. If you have any questions after this you can contact me at ______. Thank you for your time today. Goodbye.
