# Supplementary material for: Associations between the Gut Microbiota, Race, and Ethnicity of Patients with Colorectal Cancer: A Pilot and Feasibility Study
Source: Cancers (Basel). 2023 Sep 13;15(18):4546. doi: 10.3390/cancers15184546 (PMC10526839; doi:10.3390/cancers15184546)
Supplement: Supplementary file 1 [file cancers-15-04546-s001.zip › Supplementary Table S1.pdf]

Supplementary Table S1: Recruitment and Enrollment Procedures

Step[a]	Definition	Information Collected
Obtain list of potentially eligible patients	Direct referral or by reviewing schedules of visits	Name, medical record number, diagnosis, race/ethnicity
Review list of patients for preliminary eligibility	Review electronic health record to screen for potential eligibility	Reasons for ineligibility: no active disease, on antibiotics, current therapy, language, race/ethnicity
Eligible based on medical record review; attempt phone contact	Called by telephone, 3 attempts per number, kept log of phone numbers, number of contacts	Wrong number<br>Called 3 times, no answer<br>Phone answered, VM only<br>Phone answered, individual not available
Contact, explain study	Telephone recruitment script outlined type of study, reason they were contacted, brief study procedures and duration, compensation	Reasons for not being interested
Interested, screen to confirm eligibility	Screened for details of prior or current antibiotic use not in chart, details of current therapy (if any)	Reasons for ineligibility
Eligible, invite to join study	If interested, participant invited to schedule time for consent in person or via telephone	Reasons for declining study
Consent	Conducted via telephone or in clinic	n/a

[a]Several of these steps are described in supplemental figure 1.
