# Supplementary material for: Associations between the Gut Microbiota, Race, and Ethnicity of Patients with Colorectal Cancer: A Pilot and Feasibility Study
Source: Cancers (Basel). 2023 Sep 13;15(18):4546. doi: 10.3390/cancers15184546 (PMC10526839; doi:10.3390/cancers15184546)
Supplement: Supplementary file 1 [file cancers-15-04546-s001.zip › Supplementary Tables S5 and S6.pdf]

Supplementary Table S5: Microbial diversity is associated with race/ethnicity but not other covariates (Shannon diversity index, ANOVA test).

Category	Covariate	*p*-value	FDR
SES	Race/Ethnicity	**0.049**	0.56
	Sex	0.46	0.90
	Insurance	0.61	0.90
	Education	0.38	0.90
Diet	WholeGrain (% of grains that are whole grains)	0.81	0.90
	Meat	0.80	0.90
	FruitVeg (servings of fruits and vegetables)	0.68	0.90
	BMI	0.84	0.90
	Alcohol	0.21	0.73
	Fiber	0.92	0.92
Treatment	Any surgery	0.77	0.90
	Any ostomy	0.08	0.58
	Ileostomy	0.17	0.73
	Radiation	0.58	0.90

Supplementary Table S6: Microbial composition is associated with race/ethnicity and ileostomy but not other patient covariates (Bray-Curtis ordination, PERMANOVA test).

Category	Covariate	R^2	*p*-value	FDR
SES	Race/Ethnicity	**0.15**	**0.036**	0.28
	Sex	0.06	0.21	0.58
	Insurance	0.20	0.18	0.58
	Education	0.060	0.29	0.58
Diet	WholeGrain (% of grains that are whole grains)	0.15	0.94	0.94

	Meat	0.15	0.90	0.94
	FruitVeg (servings of fruits and vegetables)	0.16	0.79	0.92
	BMI	0.11	0.55	0.86
	Alcohol	0.054	0.64	0.90
	Fiber	0.062	0.34	0.60
**Treatment**	Any surgery	0.051	0.77	0.92
	Any ostomy	0.065	0.26	0.58
	Ileostomy	**0.091**	**0.040**	0.28
	Radiation	0.069	0.16	0.58
